# Advanced Imaging of Brain Metastases: From Augmenting Visualization and Improving Diagnosis to Evaluating Treatment Response

**DOI:** 10.3389/fneur.2020.00270

**Published:** 2020-04-15

**Authors:** Elizabeth Tong, Kassie Lyn McCullagh, Michael Iv

**Affiliations:** ^1^Stanford University Medical Center, Stanford, CA, United States; ^2^Neuroradiology, Stanford University Medical Center, Stanford, CA, United States

**Keywords:** cube, neural network, MRS, quantitative magnetization transfer (qMT), trans-membrane water exchange, chemical exchange saturation transfer (CEST), radiomic, artificial intelligence

## Abstract

Early detection of brain metastases and differentiation from other neuropathologies is crucial. Although biopsy is often required for definitive diagnosis, imaging can provide useful information. After treatment commences, imaging is also performed to assess the efficacy of treatment. Contrast-enhanced magnetic resonance imaging (MRI) is the traditional imaging method for the evaluation of brain metastases, as it provides information about lesion size, morphology, and macroscopic properties. Newer MRI sequences have been developed to increase the conspicuity of detecting enhancing metastases. Other advanced MRI techniques, that have the capability to probe beyond the anatomic structure, are available to characterize micro-structures, cellularity, physiology, perfusion, and metabolism. Artificial intelligence provides powerful computational tools for detection, segmentation, classification, prediction, and prognosis. We highlight and review a few advanced MRI techniques for the assessment of brain metastases–specifically for (1) diagnosis, including differentiating between malignancy types and (2) evaluation of treatment response, including the differentiation between radiation necrosis and disease progression.

## Introduction

Early detection of brain metastases (BM) and accurate differentiation from other neuropathologies is crucial. Early diagnosis affects prognosis and outcome (1). Separating metastases from other etiologies such as primary brain tumors, infection, demyelination, infarction, and hemorrhage is important because the respective treatments are vastly different. Although biopsy is often required for definitive diagnosis, imaging can provide useful information.

Recent improvements in local procedures combined with newer systemic treatments, including targeted therapeutics, have substantially modified the prognosis and survival of patients with brain metastases. Primary approaches to the treatment of brain metastases include surgery, stereotactic radiosurgery (SRS), and whole brain radiation therapy (WBRT). One key determinant in informing treatment decisions is the number of metastases present. Convergent data suggest SRS to the surgical cavity is preferable to WBRT in most patients with up to four brain metastases (2, 3), providing similar intracranial disease control with less risk of neurocognitive decline. Treatment for patients with multiple (>4) brain metastases has yet to be determined (4). Although expert opinion on the limit on number and size varies, there is no question that accurate accounting of the number of brain metastases is necessary.

Besides detection and tallying, imaging is also performed to assess treatment effects. According to the Response Assessment in Neuro-Oncology Brain Metastases (RANO-BM) working group's proposal, the size of metastases is an important criteria for assessing treatment response (5). Indeed, the four categories of response (complete response, partial response, progressive disease, and stable disease), are defined based on the lesion size. Another crucial task for clinicians and radiologists after radiotherapy is the distinction between radiation necrosis and tumor progression, which is challenging because of their overlapping features on conventional MRI sequences. Recent advances in the treatment of brain metastases (e.g., immunotherapy and targeted therapies) have also posed challenges for the interpretation of MRIs, specifically with regard to the question of pseudoprogression or radiation necrosis vs. true disease progression.

Traditionally, contrast-enhanced magnetic resonance imaging (MRI) is the preferred imaging study for the diagnosis of brain metastases (6, 7). The two most commonly used MRI sequences for assessing brain metastases are contrast-enhanced T1-weighted (CET1W) and T2-weighted FLAIR, which provide information about size, morphology and macroscopic structures. Newer MRI sequences have been developed to increase the conspicuity of enhancing metastases. More recently, advanced MRI techniques that have moved beyond anatomical imaging are available to characterize microstructures, cellularity, physiology, perfusion, and metabolism. Changes in these attributes may supersede perceivable macroscopic anatomic changes and can serve as potential biomarkers for monitoring treatment effect, recurrence, and disease progression (8).

The recent interest of artificial intelligence has transformed the field of medicine. Radiomics and deep learning are deployed to unveil discernible and grossly indiscernible features within radiological images, which can assist with decision-making in oncology (9, 10). Radiomics use sophisticated computational methods to extract quantitative features from medical images, which can be beyond human visual perception (9). A vast amount of computational data are generated, which are then mined by using various machine-learning algorithms to develop models that may potentially improve diagnostic, prognostic, and predictive accuracy (9). On the other hand, deep learning uses multilayer artificial neural networks to learn imperceptible features directly from data, without the constraints of predefined equations and is a powerful tool for classification, detection, and segmentation tasks (11).

Here, we highlight and review the utility of advanced MRI techniques, including new imaging sequences, quantitative methods, and artificial intelligence to evaluate brain metastases–specifically for (1) diagnosis, including differentiating between malignancy types and (2) evaluation of treatment response, including the differentiation between radiation necrosis and disease progression.

## Black Blood MR Imaging

A clinically dedicated brain metastasis MRI protocol typically consists of pre-contrast (i.e., diffusion-weighted, T2-weighted, T1-weighted) and post-contrast (i.e., T1-weighted, FLAIR) sequences. The critical sequence is the postcontrast 3D T1-weighted sequence, which is a high-resolution sequence acquired by either 3D volumetric Fast Spoiled Gradient-Echo (FSPGR) or Fast Spin-Echo (FSE) technique.

**Figure 1:**
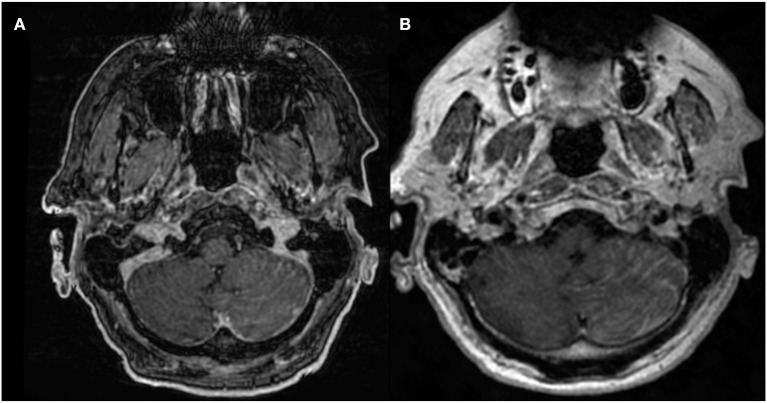
68-years-old patient with primary lung adenocarcinoma and leptomeningeal carcinomatosis. Diffuse nodular leptomeningeal enhancement is seen over the left cerebellar surface on **(A)** post-contrast 3D T1 FSPGR and **(B)** post-contrast 3D T1 CUBE, compatible with leptomeningeal carcinomatosis. Conspicuity of enhancement is increased in **(B)** compared to **(A)**.

3D volumetric gradient echo imaging (e.g., BRAVO, GE Healthcare; MPRAGE, Siemens Healthcare; 3D TFE; Philips Healthcare) is employed broadly because of the excellent gray-white matter differentiation provided by the technique. 3D volumetric FSE imaging (e.g., CUBE, GE Healthcare; SPACE, Siemens Healthcare; VISTA, Phillips Healthcare) is a relatively newer technique that is also optimal for high-resolution imaging. T1-weighted, T2-weighted, PD-weighted, or FLAIR images can be obtained with the FSE technique. One important distinguishing feature between both of these techniques is the appearance of the vessels. Specifically, the vasculature appears bright on post-contrast 3D T1 FSPGR but appears dark (“black blood”) on post-contrast 3D T1 FSE. As a result of the “bright blood” appearance on post-contrast FSPGR, it can sometimes be difficult to distinguish enhancing parenchymal metastases or leptomeningeal carcinomatosis (Figure 1) from background vascular enhancement (12). On the contrary, post-contrast FSE provides inherent background vascular suppression, yielding a higher contrast-to-noise ratio (CNR) of lesions, making enhancing parenchymal, and leptomeningeal metastases more conspicuous (13).

### Detection of Brain Metastases

**Figure 2:**
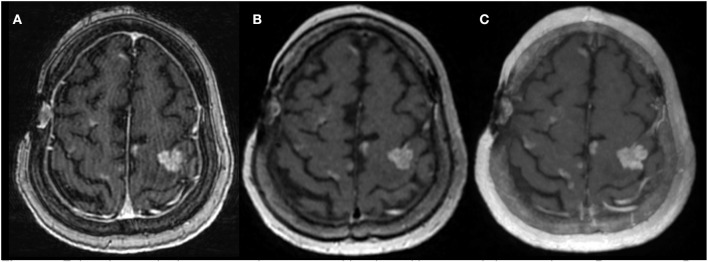
Enhancing cerebral metastases in a 78-years-old patient with metastatic lung carcinoma. Postcontrast 3D T1-weighted **(A)** FSPGR, **(B)** CUBE, and **(C)** overlapping CUBE maximum intensity projection (oC-MIP) images demonstrate enhancing metastatic lesions. The lesions appear most conspicuous with overlapping CUBE MIPs **(C)**. The contrast-to-noise ratio of lesions is also highest for oC-MIP **(C)**.

To make metastases even more perceptible, thick-section maximum intensity projection (MIP) images can be reconstructed from post-contrast 3D T1 FSE. Reconstruction with slice overlapping can further help to reduce artifacts from partial volume averaging and improve visualization of lesions (Figure 2) (14). The use of MIP images is standard practice for detecting pulmonary nodules in chest imaging (15), because discrete lesions are accentuated from the background. Yoon et al. used a similar technique and demonstrated better and faster detection of brain metastases using MIP images constructed from overlapping post-contrast T1-weighted CUBE (oCUBE-MIP). They compared oCUBE-MIP images with more conventional imaging techniques–source post-contrast 3D T1 FSPGR, source post-contrast 3D T1 CUBE, and non-overlapping CUBE MIPs (nCUBE-MIP) (14). As expected, the CNR was highest on oCUBE-MIP and lowest on FSPGR, for both small (<4 mm) and large lesions (>4 mm). The sensitivity for lesion detection was highest with oCUBE-MIP (0.96). oCUBE-MIP had a slightly higher false-positive rate than FSPGR, which they attributed to erroneous diagnosis of tiny vascular segments as punctate metastases. However, the false-positive rate of oCUBE-MIP was improved when source CUBE images were provided along with oCUBE-MIP images for cross-referencing purposes, because vessels can be more accurately traced on source CUBE images. The mean interpretation time with oCUBE-MIP was 94.7 +/– 36.5 s, which was significantly lower than with source CUBE (173.5 +/– 67.7 s) and source BRAVO (195 +/– 64.8 s) alone, providing an average saving of at least 100 s per case.

Taking advantage of the higher CNR on CUBE images, Oh et al. demonstrated significantly better diagnostic accuracy for detection of leptomeningeal carcinomatosis in 78 subjects with post-contrast 3D T1 CUBE images (*p* < 0.05). Highest sensitivity was achieved on post-contrast CUBE (97.43%), followed by post-contrast 2D T1-weighted spin-echo (66.67%), and post-contrast T1 FSPGR (64.1%). There were no significant differences in specificities among the three imaging techniques (16).

#### Clinical Implication

Post-contrast 3D T1 CUBE, particularly with overlapping thick-section MIP reconstruction, is a clinically available imaging technique that offers high contrast-to-noise ratios of enhancing lesions within the brain and allows for fast and sensitive detection of brain metastases (Table 1).

**Table 1:** Summary of imaging techniques, features, and potential applications.

**Technique**	**Features**	**Potential applications**
Overlapping post-contrast 3D T1-weighted CUBE (oCUBE-MIP)	High contrast-to-noise ratio of enhancing lesions	•Fast and sensitive detection of brain parenchymal and leptomeningeal metastases •Clinically available and easy to implement
Magnetic resonance spectroscopy	Detects tumoral metabolites	•Lipids detected in metastases and glioblastomas •Higher NAA/Cr in metastases than in primary gliomas •Higher Cho/Cr and Cho/NAA with tumor progression than with radiation necrosis •Clinically available but more difficult and complex to implement; complementary to structural imaging •Standardization across different MRI vendors is needed
Quantitative magnetization transfer	Characterizes magnetization transfer ratio (MTR), macromolecular concentration (F), exchange rate between the bound protons and free water protons (kf)	•Peritumoral MTR lowest in meningioma compared to glioblastoma and metastases •Macromolecular fraction in the non-contrast-enhancing region of tumor highest in metastases •Largely investigational at this time •No standardized post-processing software
Trans-membrane water exchange	Measures transmembrane intra-extracellular water exchange rate constant (kIE) which is sensitive to apoptosis	•kIE higher in responders (to radiosurgery) than non-responders •Largely investigational at this time
Chemical exchange saturation transfer	Measures metabolites of neoplasm milieu	•Higher MTR_Amide_ and NOE with tumor progression than with radiation necrosis. •Promising and rapidly developing molecular-imaging tool •More studies in humans and standardized techiques to improve the specificity are needed
Perfusion imaging	Relative cerebral blood volume and cerebral blood flow	•Peritumoral rCBV and rCBF higher in glioblastomas than metastases •Intratumoral rCBV can help to distinguish infection from tumor •Higher intratumoral and peritumoral ASL-rCBF in glioblastomas than in metastases. •Higher rCBV, higher rPH, lower PSR, higher Ktrans in recurrent tumor; lower rCBV, lower rPH, higher PSR, lower Ktrans in radiation necrosis •Clinically available with different acquisition and post-processing methods, limiting its universal adoption; complementary to structural imaging
Radiomics and textural analysis	Computes quantitative patterns and inter-pixel relationships of tumors	•Some textural parameters can distinguish glioblastomas from metastases Some textural parameters can classify the primary origins of brain metastases •Largely investigational at this time; large multicenter datasets are needed for validation

## Magnetic Resonance Spectroscopy (MRS)

Protons of different molecules resonate at slightly different frequencies secondary to the local magnetic field generated by the electron cloud surrounding them. Magnetic resonance spectroscopy (MRS) detects tissue metabolites by their characteristic resonant frequencies (17). In oncologic applications, the metabolites of interest are products or byproducts of malignancy-related pathways (17, 18). The most common metabolites are N-acetylaspartate (NAA), choline (Cho), lipid (Lp), and creatine (Cr). NAA is a neurotransmitter, which is abundant in neurons, and is a marker for neuronal health (19). Its concentration is related to the extent of neuronal destruction (20). Choline is involved in the manufacturing of phospholipids, which is an integral component of cell membranes (21). Higher levels of choline are associated with higher cell membrane turnover, presumably from cell damage. Lipid is a byproduct associated with cellular necrosis and is often seen in the setting of glioblastoma or metastases. Creatine is involved in intracellular metabolic processes. Creatine concentration is higher in areas with higher energy metabolism (22). The concentration of these metabolites can be measured on MRS and can help to determine the underlying pathophysiology of a lesion.

There are many spectroscopic acquisition techniques, with commonly used methods being “PRESS” (23) and “STEAM” (24). While technical details of MRS are beyond the scope of this article, MRS can be acquired with either short or long echo-time (TE), with typical short TE values ranging between 18 and 45 ms and long TE values ranging between 120 and 288 ms. Different TE values highlight different aspects of the spectra. For example, on short TE MRS, the spectra tend to have an irregular fluctuating baseline, and NAA may overlap with the glutamine/glutamate peak. On long TE MRS, lipids may not be detected, and there may be an artifactual elevation of the Cho/Cr ratio (25). The optimal TE for brain malignancy MRS is still under discussion (25). MRS is acquired by using a single voxel technique, with a small voxel size of a few cubic centimeters, or with a (2D or 3D) multi-voxel technique, which provides larger coverage of a target lesion at a higher spatial resolution. However, both methods are limited in spatial resolution and coverage, making MRS susceptible to partial volume effects. Diagnostic accuracy of MRS can also be limited by sampling error, especially with heterogeneous lesions (8). In general, a multi-voxel technique is recommended for evaluation of heterogeneous tumors or multiple lesions in order to minimize sampling error from a specific area of a lesion (26). Moreover, voxels should be positioned away from fat, bone, air, ventricles, vessels, and cerebrospinal fluid in order to avoid contamination of the spectra.

### Differentiate Malignancy Types

Similar to glioblastoma, brain metastases express elevated lipid signal (presumably as a result of cellular necrosis) on MRS. The lipid peak, therefore, has been used to differentiate these two tumor types from other brain neoplasms (27). Ishimaru et al. studied 11 patients with anaplastic gliomas, 20 patients with glioblastomas, and 25 patients with brain metastases using single-voxel MRS to differentiate between the three malignancy types (27). They measured the levels of lipids, NAA, Cho, and Cr. Metastases and glioblastomas showed definite lipid peak or lipid/lactate mixture peak, but no lipid signal was detected in anaplastic gliomas. Absence of the lipid signal precluded metastases. A strong Cho peak was detected in all tumors. No definite Cr peak was detected in 21 of the 25 metastases. Therefore, the presence of a Cr peak was suggestive of glioma, whereas the absence of a Cr peak was more suggestive of metastasis. The NAA/Cr ratio was shown to be higher in brain metastases (NAA/Cr = 1.58 ± 0.56), as compared to anaplastic gliomas (NAA/Cr = 0.70 ± 0.23) and glioblastomas (NAA/Cr = 0.76 ± 0.40) (27).

Similar results were reported in another study investigating 32 patients with high-grade gliomas, 14 patients with low-grade gliomas, and 14 patients with brain metastases using multi-voxel 2D MRS on 3T (28). Both NAA/Cr and Cho/Cr ratios within the tumors were higher in brain metastases (NAA/Cr = 4.43 ± 4.5, Cho/Cr = 4.88 ± 7.0), than in low-grade gliomas (NAA/Cr = 1.68 ± 0.9, Cho/Cr = 2.7 ± 2.1), and high-grade gliomas (NAA/Cr = 1.04 ± 0.6, Cho/Cr = 3.4 ± 1.7). In the peritumoral regions, NAA/Cr and Cho/Cr ratios were highest in low-grade gliomas (NAA/Cr = 3.73 ± 2.61, Cho/Cr = 4.62 ± 6.95), followed by brain metastases (NAA/Cr = 2.53 ± 1.13, Cho/Cr = 2.72 ± 2.55), and lowest in high-grade gliomas (NAA/Cr = 1.49 ± 0.83, and Cho/Cr = 2.49 ± 2.02) (28). Higher lipids were measured in high-grade gliomas (Lipids = 118.2 ± 215.9), which could help to discriminate them from metastases (Lipids = 35.48 ± 48.22). However, lipid levels in low-grade gliomas were similar to that of metastases and were therefore not useful to discriminate between the two (28). Lactate signal was also significantly different in high-grade gliomas (Lactate = 94.62 ± 123), with respect to low-grade gliomas (Lactate = 50.02 ± 97.89), and metastases (Lactate = 15.07 ± 16.74). Of note, the reported standard deviations in metabolite measurements were quite large and overlapped between tumor types. Therefore, further studies with larger populations are needed to better determine if MRS is useful for differentiating brain metastases from different glioma types.

### Evaluate Treatment Effect

Sjobakk et al. used single voxel MRS to study lipid peak in 21 patients with brain metastases before treatment to predict outcome. Patients with a higher lipid signal at baseline had a higher 5-months survival rate. Four patients in the cohort underwent repeat MRS after treatment, which demonstrated decreased lipid signal. The two patients with a larger drop in lipid signal survived longer than the other two (16 vs. 3 months) (29).

MRS has also been used to differentiate radiation necrosis from tumor progression in brain metastases. Weybright et al. evaluated MRS in 29 patients with brain metastases after radiotherapy. Metastases that progressed showed significantly higher ratios of Cho/Cr and Cho/NAA compared to radiation necrosis (30). Schlemmer et al. also used MRS to differentiate radiation necrosis from disease progression in 56 patients (6 metastases, 2 meningiomas, 50 grade I to IV astrocytomas). Higher Cho/Cr and Cho/NAA ratios (*p* < 0.0001) were observed in tumor progression (*n* = 34) compared with radiation necrosis or stable disease (*n* = 32) and contralateral normal brain (*n* = 33). Using Cho/Cr and Cho/NAA ratios to classify a lesion as progressive tumor yielded 82 and 81% accuracy, respectively (31).

These findings were further interrogated in a meta-analysis of 13 studies, encompassing 397 lesions, that showed higher Cho/Cr and Cho/NAA ratios in tumors than in radiation necrosis (22). There was a significant difference in Cho/Cr ratio between recurrent tumor and radiation necrosis (0.77, 95%CI = 0.57 to 0.98, *p* = 0.001). There was also a significant difference in ratios of Cho/NAA between recurrent tumor and radiation necrosis (1.02, 95%CI = 0.03 to 2.00, *p* = 0.044). However, there was a large overlap in the values between the two groups.

These promising studies suggest that the concentrations and ratios of metabolites in tumor milieu detected by MRS may be useful in distinguishing between the following groups: neoplastic and non-neoplastic brain lesions, progressive disease and radiation necrosis, and treatment responders and non-responders.

#### Clinical Implication

**Figure 3:**
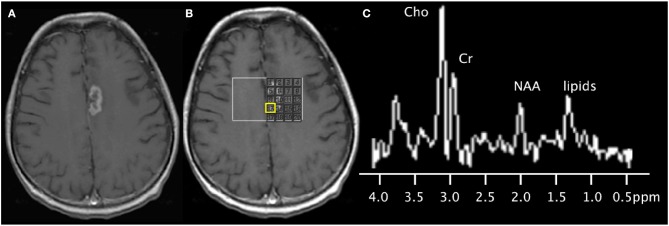
67-years-old patient with a left medial frontal lobe brain metastasis from primary lung cancer treated with stereotactic radiosurgery. **(A)** Post-contrast T1-weighted image shows the treated enhancing lesion. **(B)** Multi-voxel 2D magnetic resonance spectroscopy acquired at 3T with grid placed on the lesion. **(C)** Spectra (from the voxel highlighted in yellow) shows findings of disease progression, with the presence of a lipid peak (indicative of necrosis), high Cho peak, and increased Cho/NAA and Cho/Cr ratios. Subsequent short-term follow-up MRI showed growth of the lesion (not shown), supporting the diagnosis of disease progression.

**Figure 4:**
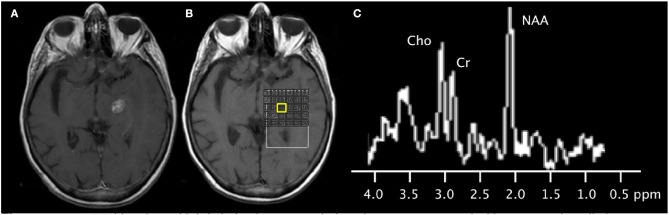
79-years-old patient with left thalamic metastasis from lung cancer treated with stereotactic radiotherapy. **(A)** Post-contrast T1-weighted image shows the treated enhancing lesion. **(B)** Multi-voxel 2D magnetic resonance spectroscopy acquired at 3T with grid placed on the lesion. **(C)** Spectra (from the voxel highlighted in yellow) shows mild elevation of the choline peak, slightly increased Cho/Cr ratio, slightly increased Cho/NAA ratio, and normal NAA/Cr ratio. Subsequent follow-up MRI showed shrinkage of the contrast-enhancing mass (not shown), supporting the diagnosis of radiation necrosis.

MRS is a clinically available technique that provides information on tumoral metabolites in the treatment naïve and post-treatment setting (Figures 3, 4). However, given overlapping features with different tumor types and subtypes and other metabolically active disease processes, prospective studies with larger sample sizes are needed to further investigate its potential diagnostic capabilities (Table 1). Partial volume effects and limited coverage are some of the reasons why MRS is replaced by other whole-brain techniques such as perfusion. Standardization across sites and different vendors of acquisition and analysis techniques is also needed before MRS can be widely adopted as clinical tool (32).

## Quantitative Magnetization Transfer (qMT)

Magnetic transfer describes the phenomenon where net magnetization of free water hydrogen protons is exchanged with that of restricted hydrogen protons (those bound to macromolecules) (33). Such macromolecules include lipids constituted in myelin and cell membranes. Magnetization transfer imaging (MTI) applies radiofrequency energy (MT pulses) to the bound protons, which is then transferred to the free water pool (34). Depending on the degree of coupling between these pools, the free water pool becomes partially saturated. When imaged, this saturation effect (secondary to magnetization transfer) manifests as signal loss. Quantitative magnetization transfer imaging (qMT) characterizes the magnetization transfer ratio (MTR), the macromolecular concentration (F), the exchange rate between the bound protons and free water protons (kf), as well as the relaxation times (T1, T2) of the bound and free proton pools (34). MTR of each voxel is computed as: MTR = (So – SMT)/So, where So is the magnitude of tissue signal before the MT pulse and SMT is the signal after applying MT pulse.

### Differentiate Malignancy Types

**Figure 5:**
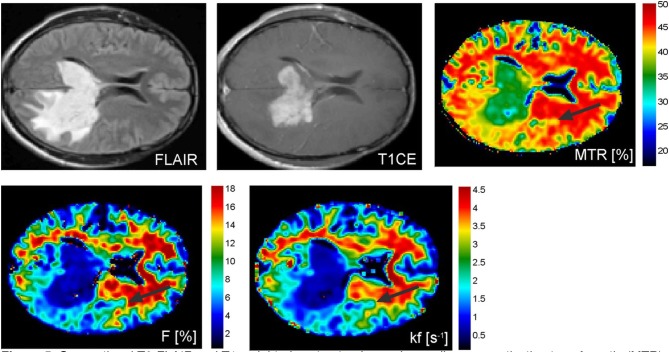
Conventional T2-FLAIR and T1-weighted contrast-enhanced as well as magnetization transfer ratio (MTR), macromolecular concentration (F), and exchange rate between the bound protons and free water protons (kf) images of a patient with a malignant pleomorphic glial tumor in the left temporal and occipital lobes showing contralateral tumor extension via the splenium of the corpus callosum. Abnormal MTR values can be discerned ventrally and laterally to the altered looking tissue on conventional MRI (black arrows). Reproduced, with permission, from Garcia et al. (35).

Garcia et al. used magnetization transfer ratio (MTR) and qMT parameters to differentiate brain metastases from other brain tumors in 26 patients (35). (Figure 5) Significant differences were found in the MTR and qMT parameters (on both the tumor rim and core) of glioblastoma, meningiomas, and brain metastases (35). MTR on the non-contrast-enhancing region of tumor was highest in metastases (MTR = 35.1% ± 0.5), followed by glioblastoma (MTR = 33.8% ± 1.2 and meningiomas (MTR = 28.9% ± 1.6), and was capable of separating metastases from meningiomas. MTR on the contrast-enhancing region was highest in meningiomas (MTR = 30.5% ± 1.2), followed by metastases (MTR = 27.4% ± 1.0) and glioblastoma (MTR = 25.2% ± 0.6), and could separate glioblastoma from meningiomas. MT exchange rate on the contrast-enhancing region of the tumor (k_f_ = 0.8 ± 0.1, 1.1 ± 0.1, 0.6 ± 0.0 for brain metastases, meningiomas, and glioblastomas, respectively) and macromolecular fraction on the non-contrast-enhancing region of the tumor (*F* = 7.2 ± 0.7, 5.6 ± 0.2, 3.6± 0.7 for brain metastases, meningiomas, and glioblastomas, respectively) could distinguish between all three tumor types (35).

#### Clinical Implication

Quantitative magnetization transfer imaging is largely research-based at this time. More and larger clinical studies are needed to validate its use in the clinical setting (Table 1). Currently, there are no FDA approved or standardized software to post-process the acquired data or display the results.

## TRANS-Membrane Water Exchange

Biologic tissue can be grossly divided into three compartments–vascular, intracellular, and extracellular extra-vascular, with different physiochemical properties. Water molecules move between these two non-vascular compartments constantly. The exchange rate of water molecules between intracellular and extracellular compartments depends on the permeability of the cell membrane as well as on the size and shape of the cell (36). Transmembrane intra-to-extracellular water exchange rate constant (kIE) is very sensitive to structure damage such as apoptosis. During apoptosis, cells are disfigured, shrunken, and have higher cell membrane permeability (36, 37), leading to an increase in kIE. It has been shown that kIE increases significantly within days after inducing apoptosis (38, 39).

### Evaluate Treatment Effect

Mehrabian et al. developed a water exchange quantification technique for dynamic contrast-enhanced MRI, to measure the transmembrane water exchange rate (kIE) (39). Since the transmembrane intra-to-extracellular water exchange rate constant (kIE) is sensitive to apoptosis, and assuming effective treatment destroys malignant cells by apoptosis, kIE can be used to distinguish responders from non-responders to therapy. The authors investigated the change in transmembrane water exchange rate (kIE) between pre-treatment and 1-week post-treatment scans, and correlated measurements with treatment efficacy in 19 patients with brain metastases undergoing stereotactic radiosurgery (39). Trans-membrane water exchange rate constant is significantly increased in responders, as determined according to RANO-BM criteria (5), than non-responders within 1 week after treatment (*p* < 0.001). In addition, the increase in transmembrane water exchange rate (kIE) correlated with tumor shrinkage at 1 month after treatment (R = −0.76, *p* < 0.001). This ability to differentiate responders from non-responders at such early post-treatment stage can potentially help to inform treatment plans.

#### Clinical Implication

Multi-center trials complying with criteria of evidence-based medicine have not yet been completed, therefore transmembrane water exchange imaging is primarily investigational at this time (Table 1).

## Chemical Exchange Saturation Transfer (CEST)

Chemical exchange saturation transfer MRI (CEST) is a novel MR technique that detects the chemical shift between exchanging protons of the metabolites with the local electron cloud (40). CEST can image certain compounds at concentrations that are too low to be detected by standard MR imaging or MRS (40–42). CEST is sensitive to the exchange of labile protons (including amide protons), rapid exchange of hydroxyl protons, and intramolecular transfer of magnetization from aliphatic (-CH) protons to labile protons, termed as relayed nuclear Overhauser effect (rNOE) (43). These protons are found in metabolites such as glutamate, lactate, myo-inositol and glucose, which are common constituents in a neoplastic milieu (44). The most commonly used CEST metrics in cancer are amide proton transfer (APT) (45), magnetization transfer ratio for amide (MTR_Amide_), and nuclear Overhauser effect (NOE) (46). NOE is the transfer of nuclear spin polarization from one nuclear spin population to another via dipolar cross-relaxation (47).

### Evaluation of Treatment Effect

**Figure 6:**
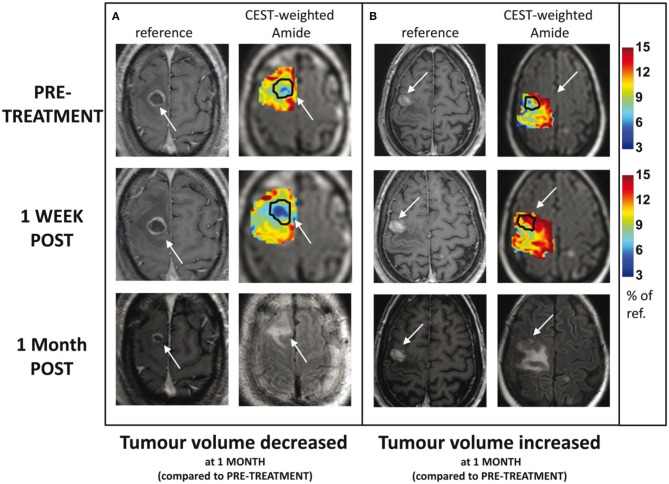
Example CEST amide effect maps within the tumor and immediately surrounding tissue, including edema, at baseline and 1 week post-treatment for 2 patients: **(A)** tumor volume decreased 1 month post-therapy and **(B)** tumor volume increased 1 month post-therapy. The margins of the enhancing tumor are indicated with an arrow and outlined in black on the CEST maps. The corresponding slice from the high resolution, contrast-enhanced T1-weighted volume is shown for comparison. Contrast-enhanced T1-weighted and FLAIR images at 1 month follow-up are shown in the third row. Reproduced, with permission, from Desmond et al. (48).

Desmond et al. applied endogenous CEST-MRI to determine response of 25 patients with brain metastases within 1 week after stereotactic radiosurgery (SRS) treatment (48). Reduced CEST signal in responders and increased CEST in non-responders were observed (Figure 6). Furthermore, changes in CEST signals at 1-week post treatment correlated with the change in tumor volume measured at 1 month post-treatment. In particular, the width of the NOE peak in tumor (correlation coefficient, *r* = −0.55, *p* = 0.028) and amplitude of NOE peak on the normal-appearing white matter (*r* = 0.69, *p* = 0.002) yielded the highest correlations (48). The amplitude of the NOE peak in the contralateral normal-appearing white matter (NAWM) at baseline (before SRS) was inversely correlated with the degree of tumor volume change at 1 month post-treatment (*r* = −0.69, *p* = 0.002), which may be an indicator of tumor aggressiveness (48).

Mehrabian et al. used CEST to differentiate radiation necrosis from tumor progression in 16 patients with brain metastases, with nine confirmed radiation necrosis and seven tumor progression (49). Both MTR_Amide_ and NOE were able to differentiate progressive tumors from radiation necrosis with high accuracy (*p* < 0.0001) (49). Higher MTR_Amide_ was measured in tumor progression (MTR_Amide_ = 12.0 ± 1.9), compared to radiation necrosis (MTR_Amide_ = 8.2 ± 1.0). Higher NOE was measured in tumor progression (NOE = 12.6 ± 1.6), compared to radiation necrosis (NOE = 8.9 ± 0.9).

#### Clinical Implication

Chemical exchange saturation transfer shows promise as a tool for molecular imaging of CNS malignancy. Although CEST is largely a research tool currently, there are rapid development in CEST techniques for improving the acquisition speed and spatial coverage (50). More studies in humans and standardized techniques to improve the specificity of the methods will be needed in order to translate into the clinical setting (50) (Table 1).

## Perfusion Imaging

**Figure 7:**
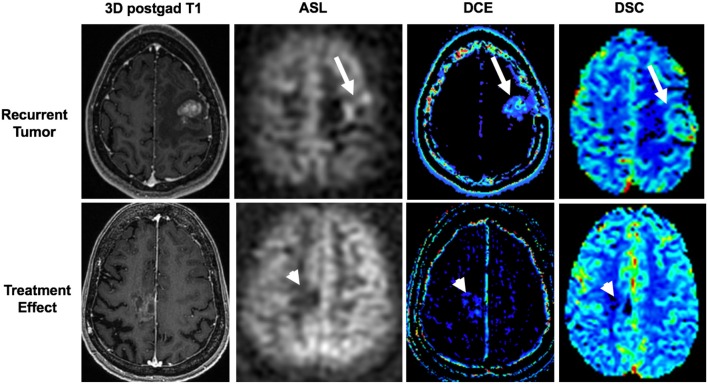
Utility of arterial spin labeling (ASL), dynamic contrast enhancement (DCE), and dynamic susceptibility contrast (DSC) perfusion MRI to differentiate between recurrent tumor and treatment effect. Top panel shows recurrent tumor in a 34-years-old female with a left frontal breast cancer metastasis that was previously resected and treated with stereotactic radiosurgery. ASL, DCE, and DSC images demonstrate high cerebral blood flow, Ktrans, and cerebral blood volume (arrows), respectively, associated with the contrast-enhancing lesion. Tumor was confirmed on histopathology from subsequent re-resection. Bottom panel shows treatment effect in a 65-years-old female with a right frontal lung cancer metastasis who was previously resected and treated with stereotactic radiosurgery. ASL, DCE, and DSC images demonstrate low cerebral blood flow, Ktrans, and cerebral blood volume (arrowheads), respectively, associated with the mildly enhancing lesion. Treatment effect was confirmed with negative PET/MRI (not shown) and a stable 3-months follow-up MRI (not shown).

The most commonly used techniques for assessing tumor perfusion are dynamic susceptibility contrast (DSC), dynamic contrast-enhanced (DCE), and arterial spin labeling (ASL) imaging (Figure 7). Different perfusion parameters are derived from each technique. For CNS tumor imaging, cerebral blood volume (CBV) and cerebral blood flow (CBF) are commonly studied metrics. CBV measures the amount of blood per volume of tissue. CBF measures the amount of blood per volume of tissue per unit of time. Both CBV and CBF reflect tumor vascularity. In addition to measuring absolute values, CBV and CBF are often measured relative to an internal control (typically the contralateral normal parenchyma). The ratios are often referred to as relative cerebral blood volume (rCBV) and relative cerebral blood flow (rCBF), respectively. In contrast to DSC and DCE imaging, ASL is a non-contrast method for determining CBF.

### Differentiation of Malignancy Types

Server et al. measured DSC perfusion parameters within the tumor and peritumoral regions to differentiate glioblastomas (*n* = 40) from metastases (*n* = 21) (51). The rCBV and rCBF within the peritumoral region were significantly higher in glioblastomas (rCBV = 1.8 ± 0.7, rCBF = 2.1 ± 1.4) than metastases (rCBV = 0.6 ± 0.1, rCBF =0.7 ± 0.5). An rCBV threshold of 0.8 yielded 95% sensitivity and 92% specificity for differentiating glioblastomas from metastasis. Other similar studies showed high negative predictive value and high specificity for detecting metastases with a peritumoral rCBV cutoff of 1.0 (52, 53).

Interestingly, the rCBV and rCBF within the tumor were not significantly different between glioblastomas and metastases (51). However, studies have shown that neoplasms (rCBV = 4.28 ± 2.11) have higher rCBV than infectious lesions (rCBV = 0.63 ± 0.49), and intratumoral rCBV can be helpful to distinguish infectious lesions from neoplasms (54).

Sunwoo et al. performed qualitative and quantitative analyses on ASL-CBF in 128 patients with glioblastoma (*n* = 89) and brain metastases (*n* = 39) (55). Intratumoral and peritumoral rCBF were assessed. Both qualitatively and quantitatively, glioblastomas demonstrated higher intratumoral and peritumoral rCBF than metastases. They report an area under the curve (AUC) of 0.835 for differentiating the two with peritumoral rCBF (55).

### Evaluation of Treatment Effect

Tumor recurrences typically develop increased abnormal vasculature, which is represented by increased rCBV. Relative peak height (rPH), which is the maximum change in signal during the passage of contrast agent, correlates with tumor capillary blood volume. Tumor recurrences will also have relatively higher rPH (56). Percentage of signal-intensity recovery (PSR), an indicator of blood-brain-barrier integrity, reflects the degree of contrast agent leakage caused by alteration of capillary permeability. Tumor recurrences often have increased permeability due to abnormally formed vessels, which allow more gadolinium to leak into and remain in the extracellular space, leading to persistent gadolinium effects of decreasing signal and consequently decreased PSR. In contrast, in radiation necrosis, the vasculature is damaged, with resulting decreased blood flow, which is represented by decreased rCBV. Also, as there is less leakage of contrast into the extracellular space, the PSR will be higher in radiation necrosis (56).

Barajas et al. used DSC perfusion imaging to aid in the diagnosis of tumor recurrence vs. radiation necrosis; the authors assessed a total of 30 lesions in 27 patients with brain metastases, which were enlarging after SRS. They showed that rCBV and rPH were statistically higher and PSR was lower in recurrent tumor than in cases of radiation necrosis. Additionally, they demonstrated that PSR was the best indicator of radiation necrosis when a cutoff value of >76.3% was used, yielding a sensitivity of 95.65% and a specificity of 100% (56).

Ktrans, derived from DCE, reflects the permeability of the tissue. An increased Ktrans suggests tumor recurrence. Morabito et al. demonstrated similar accuracy of DCE compared to DSC in distinguishing between tumor recurrence and radiation necrosis in a total of 28 patients (total of 72 lesions) in both primary brain tumors (15 cases) and metastatic lesions (57 lesions) treated with SRS. The rCBV values for DSC and the Ktrans values for DCE were compared and showed similar accuracy in differentiation radiation necrosis from tumor progression (57).

#### Clinical Implication

MR perfusion imaging is a widely available clinical technique used for assessing tumor vascularity, for differentiating between tumor types, and for differentiating tumor recurrence from treatment effect (Table 1). However, widespread adoption has been limited by lack of imaging acquisition and post-processing standardization across multiple and different institutions.

## Radiomics and Artificial Intelligence

Treatment and prognosis for patients with primary CNS malignancies and different types of metastases are different, which makes distinguishing between them important. However, these neoplastic brain lesions have overlapping features on conventional MRI, such as enhancement, surrounding edema and central necrosis. More sophisticated features, beyond standard morphometric features, are needed to distinguish them.

Texture analysis, a common radiomics approach, uses high-order statistical methods to extract quantitative patterns and inter-pixel relationships within an image. The generated computational data are then mined by using various machine-learning algorithms to develop models that may potentially improve diagnostic, prognostic, and predictive accuracy (9). Texture analysis can characterize tumor heterogeneity by evaluating relationships of gray pixels/voxels to each other using mathematical techniques such as gray-level co-occurrence matrix (GLCM), gray-level run-length matrix (GLRLM), etc. Texture analysis has been used to distinguish brain metastases from various primary malignancies (58–60).

### Differentiation of Malignancy Types

Petrujkic et al. performed texture analysis on 30 patients with glioblastomas and 25 patients with solitary metastases on T2-weighted, susceptibility weighted, and post-contrast MPRAGE (CET1) images (61). Five textural parameters were calculated–Angular second moment (S_*ASM*_), Inverse difference moment (S_*IDM*_), Contrast (S_*CON*_), correlation (S_*COR*_), and Entropy (S_*ENT*_). Compared to glioblastomas, metastases had higher *S*_*ENT*_, *S*_*COR*_, and *S*_*CON*_, and lower *S*_*ASM*_ and *S*_*IDM*_ (61). All five textural parameters from T2-weighted imaging were significantly different between glioblastoma and metastasis. Inverse difference moment (S_*IDM*_) on T2-weighted imaging was most useful for differentiating the two (sensitivity = 83.3%). On CET1 images, four textural parameters (*S*_*ASM*_, *S*_*IDM*_, *S*_*CON*_, *S*_*ENT*_) were significantly different, with Inverse difference moment (S_*IDM*_) being most specific (specificity = 84%). Performance was better when multi-sequence textural parameters were combined, achieving an AUC of 0.908, with 86.7% sensitivity and 80.0% specificity (61).

Similar results were reported in another study using perfusion imaging by Mouthuy et al. (62). Other investigators explored tumor heterogeneity by means of both 2D and 3D texture analysis in search for structural differences between brain metastases originating from different systemic cancers (63). Ortiz-Ramon et al. used random forest machine-learning approach based on texture analysis in 38 patients, to classify the primary origins of three brain metastases– lung cancer, melanoma, and breast cancer (64). Forty-three rotation-invariant 3D and 2D texture features were examined. Overall, 3D texture features were more discriminative than 2D features. A random forest classifier, using four 3D texture features, was accurate in differentiating lung cancer metastases from breast cancer metastases (AUC = 0.963 ± 0.054). Another random forest classifier, using eight 3D texture features, was very good in differentiating lung cancer metastases from melanoma metastases (AUC = 0.936 ± 0.070). However, none of their random forest classifiers were able to differentiate breast cancer metastases from melanoma metastases (AUC = 0.607 ± 0.180), presumably because of a limited small dataset. Nonetheless, texture analysis is a promising tool for classifying brain neoplasms.

### Automatic Detection and Segmentation

Typical planning in stereotactic radiosurgery (SRS) requires accurate detection and meticulous segmentation of each target lesion. Both steps are time-consuming and subject to interobserver variation. Artificial intelligence is well-suited for tackling manually tedious and repetitive tasks that require high-precision, such as brain metastases detection and segmentation. Several deep learning (DL) algorithms have been developed to detect and segment primary brain metastases, by learning from voxel-wise labeled MRI data (65–67).

**Figure 8:**
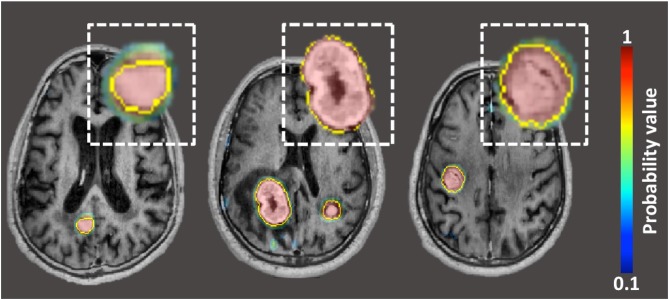
Probability maps, generated by neural-network, overlaid on post-contrast BRAVO image in patient with three lung carcinoma metastases. The yellow outline represents “ground-truth” segmentation manually drawn by neuroradiologists.

Grovik et al. designed a convolution neural network (CNN) for automatic detection and segmentation of brain metastases using multisequence (pre-contrast T1-weighted CUBE, post-contrast T1-weighted CUBE, post-contrast T1-weighted BRAVO, and 3D CUBE FLAIR) MRI data as input (68). Ground truth segmentations of the metastases were manually outlined on each slice depicting the lesion on post-contrast BRAVO images. The network's input was a slab of seven slices from each of the four sequences (pre-contrast T1-weighted CUBE, post-contrast T1-weighted CUBE, post-contrast T1-weighted BRAVO, and 3D CUBE FLAIR). The center slice of the slab was selected at the center of the metastasis. The CNN was based on the GoogLeNet architecture and was trained using TensorFlow. The output was a probability map (ranging between 0 and 1) of whether the voxel represented a metastasis (Figure 8). Voxel-wise detection accuracy was 0.98 +/– 0.04, corresponding to 94% sensitivity and 97% specificity. According to subgroup analysis and based on disease burden, the ability to detect metastatic voxels was better in patients with few (1–3) metastases than in those with more than four. Segmentation performance, as measured by the Dice coefficient, was slightly better for patients with 4–10 metastases. Using the optimal probability threshold, on a lesion-by-lesion basis, the sensitivity was 83 +/– 22%, with a false positive rate of 8.3 lesions per cases. False positive lesions were found primarily near vascular structures at the skull base such as the venous sinuses or over the cortex. Overall, the CNN performed best on patients with few metastases, both in terms of sensitivity and the number of FPs.

Other groups, using different variations of neural networks and imaging sequences as input, have reported comparable results (65–67). In essence, the CNNs have the potential to integrate detection, segmentation, and quantification of brain metastases using a streamlined process. The output of the CNN can also potentially be used as masks for radiotherapy planning.

### Evaluate Treatment Effect

Larroza et al. used texture analysis and Support Vector Machine classification (a type of machine-learning classification technique) to differentiate between brain metastases and radiation necrosis on contrast-enhanced T1-weighted images (69). A total of 179 texture features were extracted from 115 lesions from 73 patients (60 untreated lesions, 23 SRS-treated lesions, and 32 radiation necrosis). A support vector machine was used to find a subset of features that attained best classification performance. The highest classification accuracy was achieved by a machine trained with treated metastases, using a subset of 10 features (AUC = 0.94 ± 0.07). The second best performer was a machine trained with both treated and untreated metastases, using a subset of seven features and tested on treated metastases (AUC = 0.93 ± 0.02). Texture analysis on conventional MRI seems to be capable of differentiating between brain metastasis and radiation necrosis with high accuracy.

#### Clinical Implication

Studies using radiomics and machine learning in all fields of medicine are rapidly growing; however, validation with large multicenter and heterogeneous datasets is needed to confirm performance accuracy before deployment in the clinical neuro-oncology setting (Table 1).

## Conclusion

Treatment of brain metastases has become increasingly individualized as surgical and radiosurgical techniques have evolved over the past several decades. Accurate diagnosis and assessment of brain metastases in patients with systemic cancers has important implications for patient prognosis and treatment strategy. Newer anatomic imaging techniques such as “black-blood” MR imaging accentuate detection of enhancing brain parenchymal metastases and has been reported to have more sensitivity for the detection of leptomeningeal carcinomatosis. Overlapping CUBE-MIP images make identifying brain metastases easier and quicker. Advanced MRI techniques that penetrate beyond macrostructures of brain metastases, such as MRS, MR perfusion, CEST, and qMT, provide quantitative parameters that are sensitive to underlying tissue microstructure and pathophysiology. These parameters may hold promise as imaging biomarkers for monitoring disease progression and predicting treatment outcome. However, it is important to note that some of the MRI techniques highlighted in this review are still largely research-based tools and have not been integrated into clinical practice. Systematic validation using standardized protocols in the clinical setting is needed before any potential efficacy or utility of these methods is realized.

Artificial intelligence can enhance assessment of brain metastases. Texture analysis computes a large amount of intrinsic features for quantitative comparisons. Various machine-learning algorithms can be applied in tandem to extract useful features for classification. Deep Learning, utilizing neural networks, can automate detection and segmentation with high accuracy and precision.

These important advancements are helpful for promoting individualized risk-stratification, tumor characterization, and treatment decisions. However, further investigations are needed to standardize these advanced techniques and measurements. Larger multicenter clinical trials are also imperative to fully evaluate the clinical utility of these various techniques and image data.

## Author Contributions

ET was the first author and was responsible for the design, draft, figures, and review of the article. KM was the second author and was responsible for the draft and review of the article. MI was the senior author and was responsible for the design and review of the article.

### Conflict of Interest

The authors declare that the research was conducted in the absence of any commercial or financial relationships that could be construed as a potential conflict of interest.
